# Pathological patterns of TDP‐43 in LATE, Alzheimer’s Disease and FTLD‐TDP

**DOI:** 10.1002/alz.091232

**Published:** 2025-01-03

**Authors:** Sandra O. Tomé, Klara Gawor, Rik Vandenberghe, Peter T Nelson, Dietmar Rudolf Thal

**Affiliations:** ^1^ Laboratory for Neuropathology, KU Leuven, Leuven Belgium; ^2^ University Hospitals Leuven, Leuven Belgium; ^3^ KU Leuven, Leuven Belgium; ^4^ Sanders‐Brown Center on Aging, University of Kentucky, Lexington, KY USA; ^5^ UZ Leuven, Leuven Belgium

## Abstract

**Background:**

Limbic‐predominant age‐related TDP‐43 encephalopathy (LATE) has been recently recognized as a cause of dementia in the elderly. LATE and Alzheimer’s disease (AD) share similar clinical presentations, and their neuropathological changes—LATE‐NC and ADNC—commonly co‐occur in the brains of individuals with dementia. Frontotemporal degeneration (FTLD‐TDP) represents another group of TDP‐43‐associated neurodegenerative diseases. Still, the morphological and molecular features of TDP‐43 lesions in different diseases remain under‐researched. Here, we investigated distinct TDP‐43 lesions and immunoreactive species in cases of AD with LATE‐NC, pure LATE (ABC score < 1), and FTLD‐TDP.

**Method:**

We performed immunohistochemistry in paraffin‐embedded tissue from the hippocampus, amygdala, temporal and frontal cortices of 22 non‐demented cases, 24 AD cases with comorbid LATE‐NC, 9 pure LATE and 22 FTLD‐TDP cases (subtypes A‐C). We semiquantitatively assessed the severity of neuronal cytoplasmic inclusions and dystrophic neurites using antibodies against different TDP‐43 epitopes: pS409/pS403, pS403/pS404 and non‐phosphorylated C‐ and N‐terminal TDP‐43. We also assessed general neuropathological parameters among the groups.

**Result:**

LATE cases were older at death and exhibited lower neuronal density in the hippocampus compared to the remaining groups, supporting the hypothesis of a slower disease course in LATE compared to AD. AD and LATE did not differ in LATE‐NC stages.

Morphologically, pure LATE cases showed higher severity levels of hippocampal dystrophic neurites compared to AD with LATE‐NC and FTLD‐TDP cases. The severity of neuronal cytoplasmic inclusions was similar among groups. AD cases showed an overall predominance of pS409/pS410 TDP‐43 species, however LATE and FTLD‐TDP cases showed no significant differences in burden of all TDP‐43 species except N‐terminal TDP‐43, which showed lower severity in all groups. LATE cases also exhibited decreased burdens of TDP‐43 species in the frontal cortex compared to FTLD‐TDP.

**Conclusion:**

These results suggest that TDP‐43 assumes distinct patterns of immunoreactivity in dementia, especially in the hippocampus. LATE and FTLD‐TDP cases showed a similar, but non‐identical patterns of TDP‐43 immunoreactive species, patterns which were different in turn from LATE‐NC in ADNC. These data may also support the concept of LATE as a separate disease with high burdens of different TDP‐43 species restricted to the limbic system in the absence of moderate‐high ADNC.

